# LINC01980 promotes the malignant progression of lung squamous carcinoma by targeting miR-204-3p

**DOI:** 10.1186/s41065-025-00617-y

**Published:** 2025-12-13

**Authors:** Man Zhang, Weiwei Sun, Zhansheng Jiang, Zhanyu Pan, Zhuchen Yan, Lujun Zhao

**Affiliations:** 1 Department of Integrative Oncology, Tianjin Key Laboratory of Cancer Prevention and Therapy, Tianjin Medical University Cancer Institute & Hospital, National Clinical Research Center for Cancer, Tianjin’s Clinical Research Center for Cancer, 300060 Tianjin, China; 2 Department of Integrative Oncology, Tianjin Cancer Hospital Airport Hospital, 300000 Tianjin, China; 3 The Jingxi Medical District of The PLA General Hospital, Beijing, 100043 China; 4 Department of Radiation Oncology, Tianjin Key Laboratory of Cancer Prevention and Therapy, Tianjin Medical University Cancer Institute & Hospital, National Clinical Research Center for Cancer, Huanhu West Road, North of the Gymnasium, Hexi District, Tianjin, 300060 China; 5 Department of Integrative Oncology, Tianjin Key Laboratory of Cancer Prevention and Therapy, Department of Integrative Oncology, Tianjin Medical University Cancer Institute & Hospital, National Clinical Research Center for Cancer, Tianjin’s Clinical Research Center for Cancer, Tianjin Cancer Hospital Airport Hospital, No.99, Dongwu Road, Dongli Airport Economic Zone, Tianjin, 300000 China

**Keywords:** LUSC, LINC01980, MiR-204-3p, Malignant biological behavior, Clinical data

## Abstract

**Background:**

Lung squamous cell carcinoma (LUSC) is a prevalent and aggressive subtype of lung cancer. Although LINC01980 has been implicated in the development of several cancers, its specific mechanisms in LUSC remain unclear.

**Methods:**

Through GEO database analysis, LINC01980 was identified as aberrantly expressed in LUSC. Using LncBook 2.0, we predicted a target relationship between LINC01980 and miR-204-3p. Validation was performed using dual luciferase reporter assay and RNA pull-down experiments. An analysis of LUSC clinical samples and TCGA data revealed differential expressions of LINC01980 and miR-204-3p. The expression levels of these two molecules were quantified by RT-qPCR in both patient tissues and cell lines. To measure cell proliferation, the CCK-8 assay was applied. Apoptosis was evaluated via flow cytometry, whereas cell migration and invasion were investigated using the Transwell assay. Gene knockdown experiments demonstrated that LINC01980 influences malignant phenotypes in vitro by regulating miR-204-3p.

**Results:**

LINC01980 was abundantly expressed in LUSC tissues and cell lines and significantly enhanced malignant activities including proliferation, migration, and invasion. Furthermore, LINC01980 promotes tumor progression by suppressing miR-204-3p expression. Rescue experiments confirmed that the depletion of miR-204-3p reverses the tumor-suppressive effects caused by LINC01980 knockdown, indicating that the oncogenic function of LINC01980 is dependent on its negative regulation of miR-204-3p.

**Conclusion:**

LINC01980 drives LUSC invasiveness by targeting miR-204-3p, providing new insights into its tumor-promoting role and underlying mechanisms.

## Introduction

In terms of both rapid progression and fatality, few cancers match the severity of lung cancer on a global scale [1, 2]. As a major subtype of non-small cell lung cancer (NSCLC), lung squamous cell carcinoma (LUSC) accounts for approximately 20–30% of all lung cancer cases [3]. Research indicates that the five-year survival rate of patients with LUSC remains consistently poor [4]. Moreover, due to the subtlety of early symptoms, a significant proportion of patients are not diagnosed until the illness has progressed to an intermediate or advanced level, resulting in limited treatment options and poor prognosis [5]. Given this, a deeper exploration of the molecular drivers of squamous cell carcinoma formation is essential.

Exceeding 200 nucleotides in size, long noncoding RNAs (lncRNAs) function as mediators of gene expression. They contribute to cancer development through mechanisms such as chromatin structure regulation and sequestration of microRNAs [6]. Recently, new research underscored the critical role of lncRNAs in the formation and worsening of lung cancer [7]. For example, PITPNA-AS1 expression levels are significantly upregulated in exosomes from lung cancer patients [8]. LINC00152 binds competitively to miR-16-5p and promotes lung adenocarcinoma (LUAD) growth [9]. PRKCQ-AS acts as a sponge for miR-582-3p and promotes LUAD progression [10]. Emerging research indicates that LINC01980 exhibits abnormal expression levels in LUSC. According to a study by Sun et al., this lncRNA shows significant upregulation in LUSC tissues [11]. Further supporting this finding, Zhang et al. demonstrated a correlation between elevated LINC01980 levels and unfavorable clinical outcomes [12]. Furthermore, LINC01980 may act as a miRNA sponge to regulate the development of LUSC. As key gene expression regulators, miRNAs usually exert their biological functions by interacting with the 3’-UTR regions of mRNAs. In LUSC, several studies have demonstrated that a variety of miRNAs exert tumor-suppressive oroncogenic functions. For example, miR-150-5p can target multiple oncogenes, thereby significantly reduceing the invasiveness of LUSC cells [13].

Research indicates that deletions involving the 3p chromosome region occur at a higher frequency in lung cancer [14]. Notably, miR-204-3p has been implicated in the progression of NSCLC, primarily through its modulation of the miR-204-3p/ERBB2 signaling pathway [15]. Bioinformatics analyses have characterized a potential miR-204-3p target sequence within LINC01980. Despite this finding, the expression patterns of LINC01980 in LUSC patients, as well as the functional consequences of its interaction with miR-204-3p on tumor progression, remain poorly understood.

Based on the above, we hypothesized that altered LINC01980 expression in LUSC could affect tumor cell proliferation, migration, or invasion by regulating miR-204-3p levels. To validate these hypotheses, we examined the expression profiles of LINC01980 and miR-204-3p in clinical samples and in vitro cultured LUSC cells and their regulatory role in the malignant phenotype of LUSC cells. This study could provide new targets for precision therapy of LUSC.

## Materials and methods

### Patient-derived tissue samples

For this study, we selected 65 patients with a final histologically confirmed diagnosis of LUSC who underwent surgical treatment at the Tianjin Cancer Hospital Airport Hospital from June 2021 to August 2023. For each patient, neoplastic tissue and paired distal non-cancerous lung tissue (≥ 5 cm from the tumor edge) were collected during surgery. The tissues were then dissociated, flash-frozen in liquid nitrogen, and stored in a −80℃ refrigerator. It is particularly important to note that none of the patients in this study had received any anti-cancer treatment, such as radiotherapy or chemotherapy, before surgery. Clinical indicators and follow-up data were collected for later analysis. Ethical clearance for the study was granted by the institutional review board at Tianjin Cancer Hospital Airport Hospital, and all subjects gave their written consent before participation. The experimental procedures were strictly followed according to the ethical framework provided by the Declaration of Helsinki.

### Cell culture and transfection

The cell material used in the experiments was provided by the Shanghai Institute of Biological Sciences (SIBS, China) and included the human bronchial 16HBE (an epithelial cell line) in addition to the squamous cell carcinoma lines SK-MES-1 and NCI-H226. Cell cultures grew in a DMEM medium containing 10% fetal bovine serum (FBS) and 1% penicillin-streptomycin antibiotic mixture. To monitor cell health, the MycoAlert™ mycoplasma detection assay (Lonza, USA) was applied to check for contamination. The entire culture process was carried out in a standard incubator with environmental parameters set at a constant temperature of 37 °C and a CO₂ concentration of 5%.

All cells were cultured for at least 24 h before transient transfection. When cell confluence reached 70%, the following substances were introduced into target cells using Lipofectamine^®^ 2000 transfection reagent (Invitrogen, USA) according to the procedure recommended in the reagent’s user manual: LINC01980-specific short hairpin RNAs (sh-LINC01980#1 and sh-LINC01980#2), miR-204-3p mimics, miR-204-3p inhibitors, and their corresponding controls. Upon completion of the six-hour transfection period, cells were moved to a 37 °C environment, and the medium was replaced with a fresh complete medium. They were then incubated for 24 h, and the effects of transfection were assessed.

### Cell viability assay

Upon completion of transfection, 5,000 cells from every group were evenly dispensed into individual wells of 96-well plates. Measurements were taken every 24 h for 4 days. 10 µL of CCK-8 assay working solution (Sunshine Biotechnology, China) was added to each well according to the reagent instructions. Plates were then transferred to an incubator maintained at a steady 37℃ and incubated for 120 min. Absorbance values in each well were detected at 450 nm using an enzyme labeling instrument (Bio-Rad, USA).

### Cell apoptosis assay

Forty-eight hours after completion of cell transfection, samples were first digested with trypsin and separated by centrifugation to obtain cell precipitates, which were then washed two to three times with phosphate buffer solution (PBS) pre-chilled to 4 °C. To measure cell death, we used the Pharmingen™ Annexin V assay kit from BD Biosciences, and all experimental steps were carried out according to the standard protocol provided by the manufacturer. Finally, analyses were performed using a FACSVantage flow cytometry system from BD Biosciences, San Jose, CA, USA.

### Transwell migration/invasion assay for evaluating cell movement

Two days post-transfection, the cells underwent lysis, were collected by centrifugation. Afterward, the cells were re-dispersed in serum-free medium, reaching a concentration of 1 × 10⁵ cells per milliliter. The experiment was performed as follows: A 10% fetal bovine serum medium was introduced into the lower compartment, while the upper chamber was filled with a cell suspension of the same density (1 × 10⁵ cells/ml, prepared in serum-free DMEM). Cells were incubated for 10 h in a thermostatically controlled 37 °C environment. Thereafter, the upper chamber was freed of non-migrating cells, and the migrated cells in the lower chamber were dyed with 0.1% crystal violet solution for 20 min (temperature maintained at 37 °C). After staining, the cells were observed under a light microscope (Nikon, Japan, 100x magnification). In five different fields selected at random, the cells were enumerated and averaged. Specific operations for the Transwell invasion assay were as follows: first, thawed Matrigel was refrigerated at 4 °C for a full night before dilution in serum-free culture medium. A total of 50 µL of the diluted Matrigel was pipetted and evenly spread on the bottom of the Transwell upper chamber. The upper chamber was loaded with 100 µL of serum-free DMEM containing 1 × 10⁵ cells, while the lower well was filled with DMEM enriched with 10% serum. After incubating the cells in a 37 °C incubator for 18 h, the upper membrane’s non-invading cells were eliminated with a moist cotton swab, and the cells that traversed to the lower membrane underwent fixation, staining, and the number of invaded cells was counted under a higher-power microscope.

### Subcellular fractionation

Cytoplasmic and nuclear RNA components from SK-MES-1 and NCI-H226 cells were isolated using RNA extraction reagents supplied by Norgen (Belmont, CA, USA) to detect LINCO1980 lncRNA expression levels. GAPDH and U6 were utilized as internal controls for cytoplasmic and nuclear RNA, respectively.

### Dual luciferase-based gene reporter assay

A binding site for miR-204-3p is present in the 3’-UTR of LINC01980. Two distinct sequences—the wild-type (WT) with an intact binding site and the mutant (MUT) with a deleted binding region—were amplified by PCR. The amplified WT and MUT fragments were then cloned into the pmirGLO vector (Promega, China). For luciferase assays, SK-MES-1 cells were plated in 24-well plates and co-transfected for 48 h with either miR-204-3p mimics or negative control (NC) mimics, together with the corresponding reporter plasmids, utilizing Lipofectamine 2000 as the transfection reagent. After the incubation period, alterations in relative luciferase activity were assessed with the Dual-Luciferase Reporter Gene Assay Kit (Promega, USA).*RNA purification and subsequent RT-qPCR quantification*.

Total RNA from the cells and tissues was isolated and purified using a pre-chilled TRIzol LS extraction reagent (Invitrogen, USA) following the supplier’s directions. Takara PrimeScript RT kit (TaKaRa, Japan) was used to synthesize cDNA from the obtained RNA samples by reverse transcription, followed by real-time quantitative PCR using SYBR Premix Ex Taq (Qiagen, USA). The relative expression levels of the target genes were calculated by the 2^−ΔΔCt^ algorithm, and the data were normalized by using GAPDH or U6 snRNA as reference controls for normalization.

### RNA pull-down assay

Following the manufacturer’s instructions, we examine the interaction between LINC01980 and miR-204-3p via RNA pull-down. Cell protein extracts were mixed with biotinylated LINC01980 RNA probes and incubated with streptavidin-coated magnetic beads for 1 h. Finally, RNA bound to the magnetic beads was extracted using TRIzol reagent (Invitrogen, USA). After reverse transcription to synthesize cDNA, the enrichment level of miR-204-3p was detected via RT-qPCR.

### Bioinformatics analysis

This study utilized the GEO database to analyze the GSE138172 dataset. This dataset comprises RNA sequencing data derived from five pairs of lung cancer tissues and their adjacent non-tumor tissues obtained from patients with NSCLC. Differential expression of lncRNAs was visualized using the Limma package in R, with the screening criteria set at a FDR of < 0.05 and an absolute log2 fold change (|log2(FC)|) of > 1.5. Furthermore, the expression level of LINC01980 was analyzed in samples from TCGA, which included both normal lung tissues and lung cancer tissues, using the UALCAN database. Additionally, a predictive analysis of the potential targets of LINC01980 was conducted using the LncBook 2.0 database.

### Statistical analysis

Each experiment was conducted with three separate replicates. Numerical results presented in the figures and tables are shown as mean values accompanied by standard deviations (mean ± SD). For statistical evaluation, SPSS software was employed, whereas GraphPad served as the tool for generating graphical representations. Depending on the study design, intergroup differences were assessed using either Student’s t-test or one-way ANOVA. A significance level of 0.05 was used to evaluate statistical relevance.

## Results

### Aberrant LINC01980 expression is observed in the tumor samples of LUSC patients

**Fig. 1:**
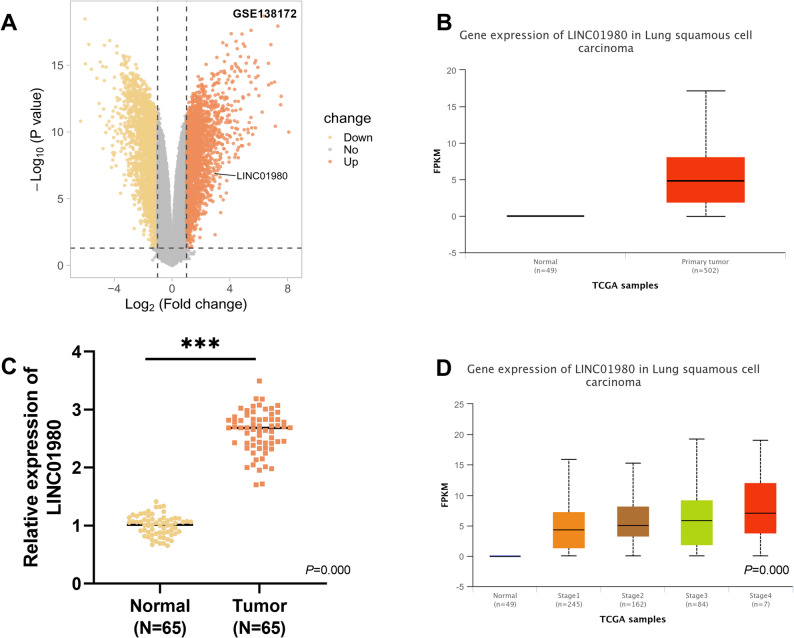
The cancer tissues of LUSCpatients show dysregulated LINC01980 expression. **A**. Differential mRNA expression volcano plot derived from the GEO database, comparing LUSC tissues with normal tissues. **B**. Relative expression of LINC01980 is significantly upregulated in LUSC tissues compared to normal tissues in the GEO dataset. **C**. Expression levels of LINC01980 in LUSC in the TCGA dataset (comparing normal and cancerous tissues). **D**. Expression levels of LINC01980 in patients with different stages of LUSC in the TCGA dataset. *** *P* < 0.001

Throughout this investigation, we first systematically searched for lncRNAs that are aberrantly expressed in lung cancer tissues. Based on the GSE138172 dataset, our analysis showed that LINC01980 was abnormally up-regulated in LUSC tissues (Fig. 1A). Analysis of the TCGA database found that LINC01980 expression in LUSC samples was significantly higher than in normal control tissues (Fig. 1B). To confirm this result, tumor tissues from 65 LUSC patients and their matched normal tissues were examined by RT-qPCR, and our findings revealed that LINC01980 expression was substantially increased in cancerous tissues (*P* < 0.001, Fig. 1C). Moreover, the expression level of LINC01980 in the LUSC samples showed a strong correlation with tumor stage (Fig. 1D). Further studies divided patients into high- and low-expression groups based on the median LINC01980 expression level. The results revealed that elevated LINC01980 expression correlated with augmented tumor dimension, nodal metastatic spread, and advanced TNM stage (III). However, no notable variations were detected in baseline demographic factors, including gender, age, and smoking history (*P* < 0.05, Table 1).

**Table 1:** Correlation between LINC01980 levels and clinicopathological characteristics of LUSC patients

Variant	LINC01980 levels		*P*
	High (*n* = 38)	Low (*n* = 27)	
Gender			0.614
Male	19	16	
Female	19	11	
Age			0.800
< 60	22	14	
≥ 60	16	13	
Smoking history			0.575
No	18	13	
Yes	20	14	
Tumour size (cm)			0.005*
≤ 5	14	20	
> 5	24	7	
Lymph node metastasis			0.044*
No	15	18	
Yes	23	9	
TNM stage			0.047*
I-II	14	17	
III	24	10	

Note: *LUSC* Lung squamous cell carcinoma. The values are shown as Number and analyzed using Chi-square test. *P* < 0.05 indicates a statistically significant difference in the data

### LINC01980 shows significant fluctuations in transcript levels in LUSC cells

**Fig. 2:**
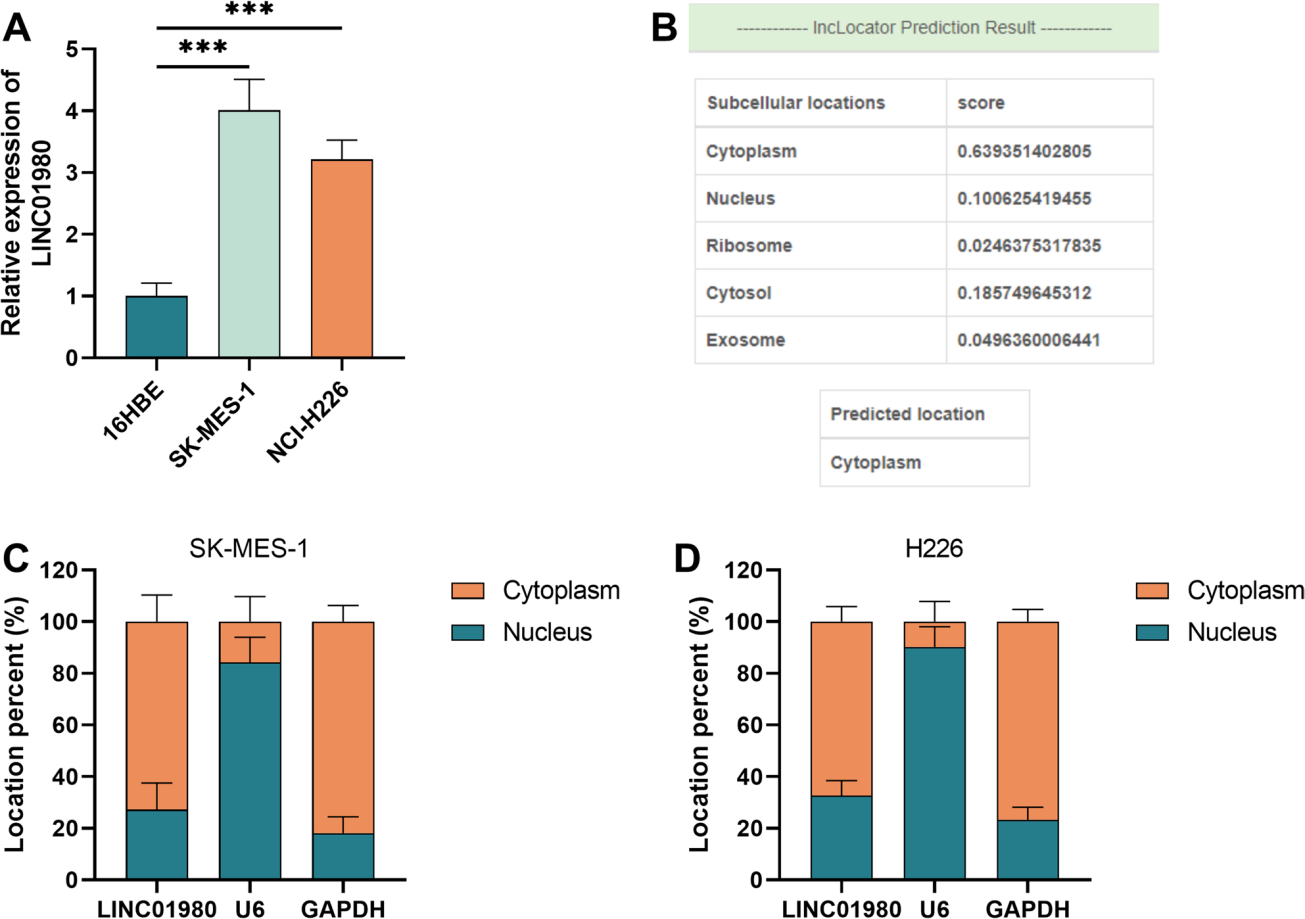
LINC01980 exhibits abnormal expression levels in LUSC cells. **A**. The expression level of LINC01980 was abnormally increased in LUSC cell lines compared to non-cancerous cells. **B**. Prediction of LINC01980 localization in cells using the LncLocator database. **C**-**D**. LINC01980 was determined to be present in the cytoplasm of LUSC cells by subcellular fractionation assay. *** *P* < 0.001

LINC01980 expression was significantly higher in LUSC cells (SK-MES-1 and NCI-H226) than in non-cancerous 16HBE cells, with the greatest increase in the SK-MES-1 cell line (*P* < 0.001, Fig. 2A). Predictive analysis of subcellular localization using the LncLocator database indicated that LINC01980 was predominantly distributed in the cytoplasm (Fig. 2B). Subsequent subcellular fractionation experiments confirmed this prediction and demonstrated that LINC01980 was indeed enriched in the cytoplasmic fraction of LUSC cells (Fig. 2C-D).

### Suppressing LINC01980 expression can effectively restrain the malignant progression of LUSC cells

**Fig. 3:**
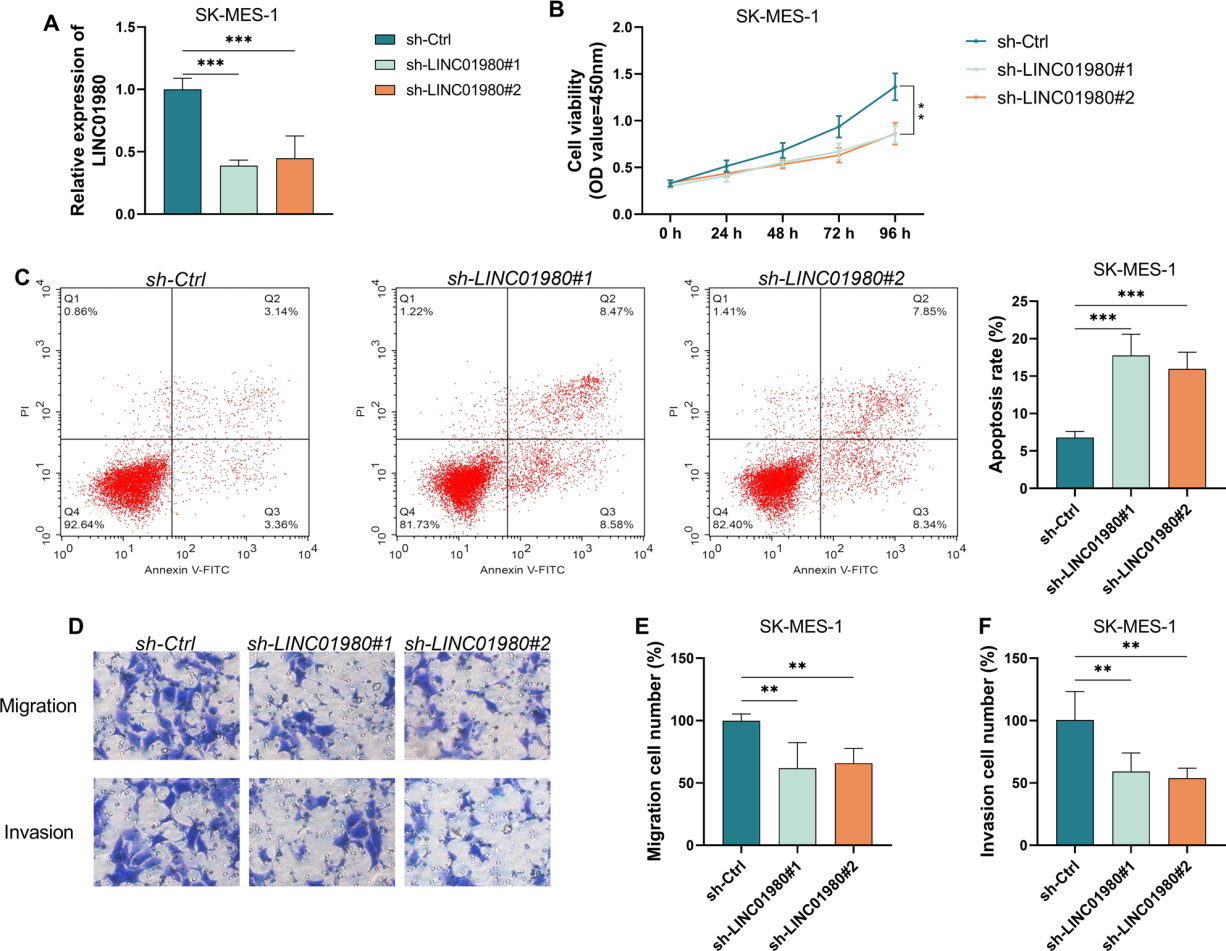
Downregulation of LINC01980 will inhibit the malignant biological behavior of lung squamous carcinoma cell lines. **A**. Assess the knockdown efficiency of sh-LINC01980#1 and #2 via RT-qPCR. **B**. Effect of LINC01980 knockdown on the proliferation of lung squamous cell carcinoma cells. **C**. Apoptosis assay and statistical analysis of apoptosis rates in SK-MES-1 cells under different knockdown conditions. **D**. Transwell assay evaluating the migration and invasion abilities of LUSC cells following LINC01980 knockdown. **E**-**F**. Migration and invasion rates in SK-MES-1 cells assessed by Transwell assay under different LINC01980 knockdown conditions. ** *P* < 0.01, *** *P* < 0.001

Since LINC01980 showed the most obvious increase in expression in the SK-MES-1 cell line, we designed two sets of short hairpin RNAs (sh-LINC01980#1 and sh-LINC01980#2) that specifically target LINC01980 to suppress its expression level. Experimental data showed that both interference vectors were effective in reducing LINC01980 levels (*P* < 0.001, Fig. 3A). Further studies showed that the inhibition of LINC01980 expression significantly reduced the proliferative activity of SK-MES-1 cells (*P* < 0.01, Fig. 3B), while the rate of apoptosis increased significantly (*P* < 0.001, Fig. 3C). Transwell analyses confirmed that interfering with LINC01980 expression significantly attenuated the migratory and invasive capacity of LUSC cells (*P* < 0.01, Fig. 3D-F). Comparing the effects of the two interference vectors, we found that sh-LINC01980#1 exhibited greater efficiency in gene silencing, so we ultimately chose sh-LINC01980#1 as the target for our subsequent experiments and formally named it sh-LINC01980.

### miR-204-3p acts as a downstream target of LINC01980 in LUSC

**Fig. 4:**
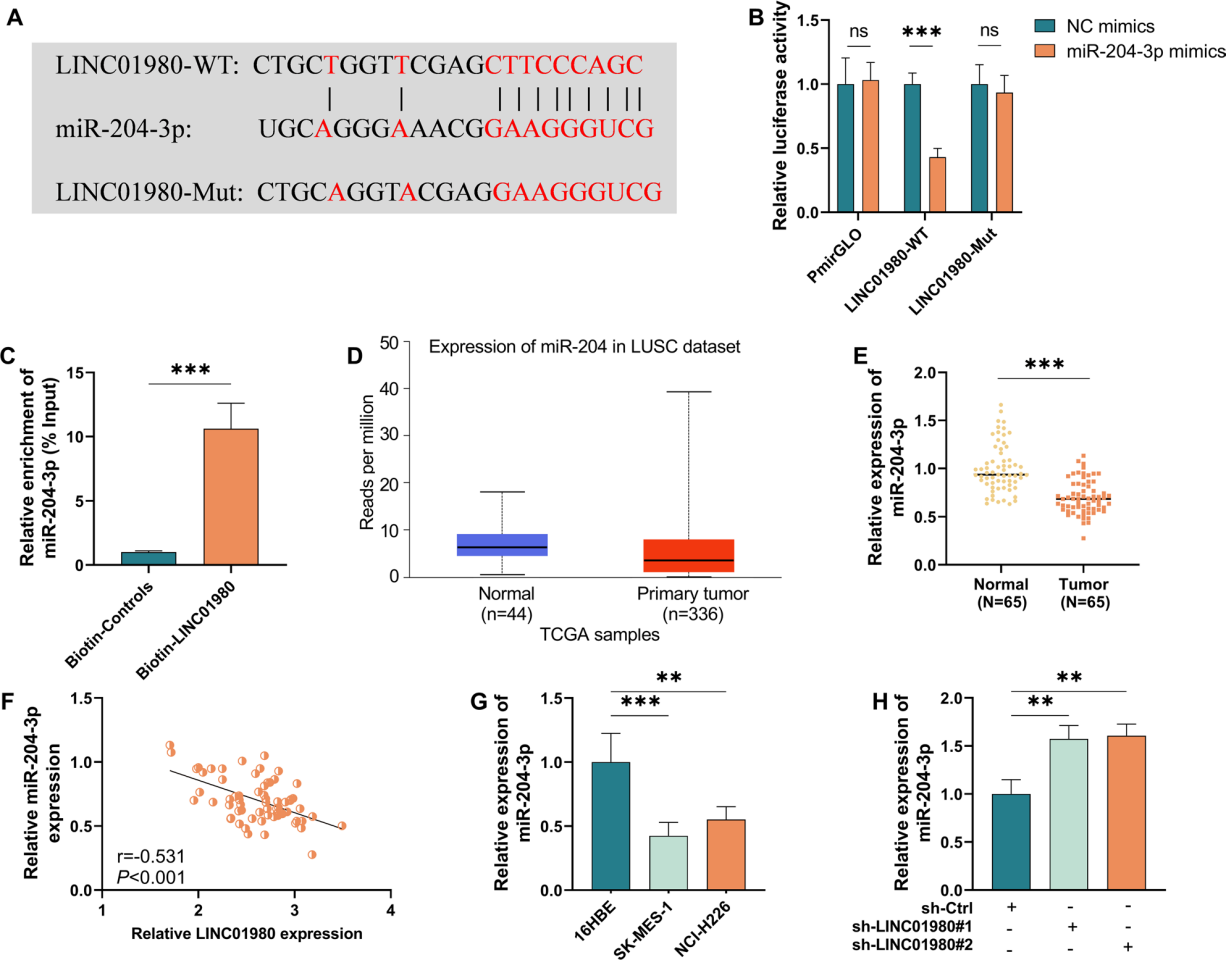
MiR-204-3p is a downstream regulatory target of LINC01980 in squamous lung cancer. **A**. Predicted binding site between miR-204-3p and LINC01980. **B**. Dual luciferase reporter assay to assess their targeting relationship. **C**. Two-targeting relationship assessed using RNA pull-down assay. **D**. Expression of miR-204-3p in neoplastic tissues of patients with squamous lung cancer employing RT-qPCR. **E**. Expression level of miR-204-3p in LUSC in TCGA dataset (comparing normal and cancerous tissues). **F**. Pearson correlation between miR-204-3p and LINC 01980 based on 65 LUSC samples. **G**. Expression of miR-204-3p in lung squamous carcinoma cell lines was detected by qRT-PCR. **H**. LUSCcells treated with miR-204-3p inhibitor were subjected to qRT-PCR to detect the expression level of miR-204-3p. ** *P* < 0.01, *** *P* < 0.001

Based on subcellular localization analysis, we have demonstrated that LINC01980 is predominantly present in the cytoplasm. This spatial distribution feature implies that the molecule may act through sponge adsorption of miRNAs. With the help of bioinformatics prediction from the LncBook 2.0 database, and based on the results, miR-204-3p was selected as a viable target (Fig. 4A). To verify this prediction, we constructed reporter gene vectors containing natural binding sequence (LINC01980-WT) and mutated sequence (LINC01980-MUT), respectively, and luciferase assay data showed that the wild-type vector showed specific inhibition of fluorescence signals when transfected with miR-204-3p mimics, whereas the mutant vector showed no significant differences between different treatment groups (*P* < 0.001, Fig. 4B). Subsequent RNA pull-down experiments further confirmed that biotin-labeled LINC01980 was able to significantly enrich miR-204-3p (*P* < 0.001, Fig. 4C). Data mining from the TCGA database for LUSC cases demonstrated that the expression level of miR-204-3p in tumor tissues showed a significant decrease compared with normal adjacent tissue samples (*P* < 0.0001, Fig. 4D). This differential expression was also confirmed in clinical samples. Using RT-qPCR, we found that the expression of this microRNA in LUSC patient tumor tissues was markedly lower when contrary to control samples (*P* < 0.0001, Fig. 4E). Expression correlation analysis noted a significant negative correlation between miR-204-3p and LINC01980 in tumor tissues (*r*=−0.531, *P* < 0.0001, Fig. 4F). Abnormally low expression of miR-204-3p was likewise detected in cellular-level studies (*P* < 0.01, Fig. 4G). Notably, following shRNA interference for LINC01980 expression reduction, a significant rebound in miR-204-3p levels was detected (*P* < 0.01, Fig. 4H). Taking the above experimental results together, we can conclude that miR-204-3p is a downstream target molecule directly regulated by LINC01980.

### LINC01980 influences malignant progression by suppressing miR-204-3p expression in LUSC

**Fig. 5:**
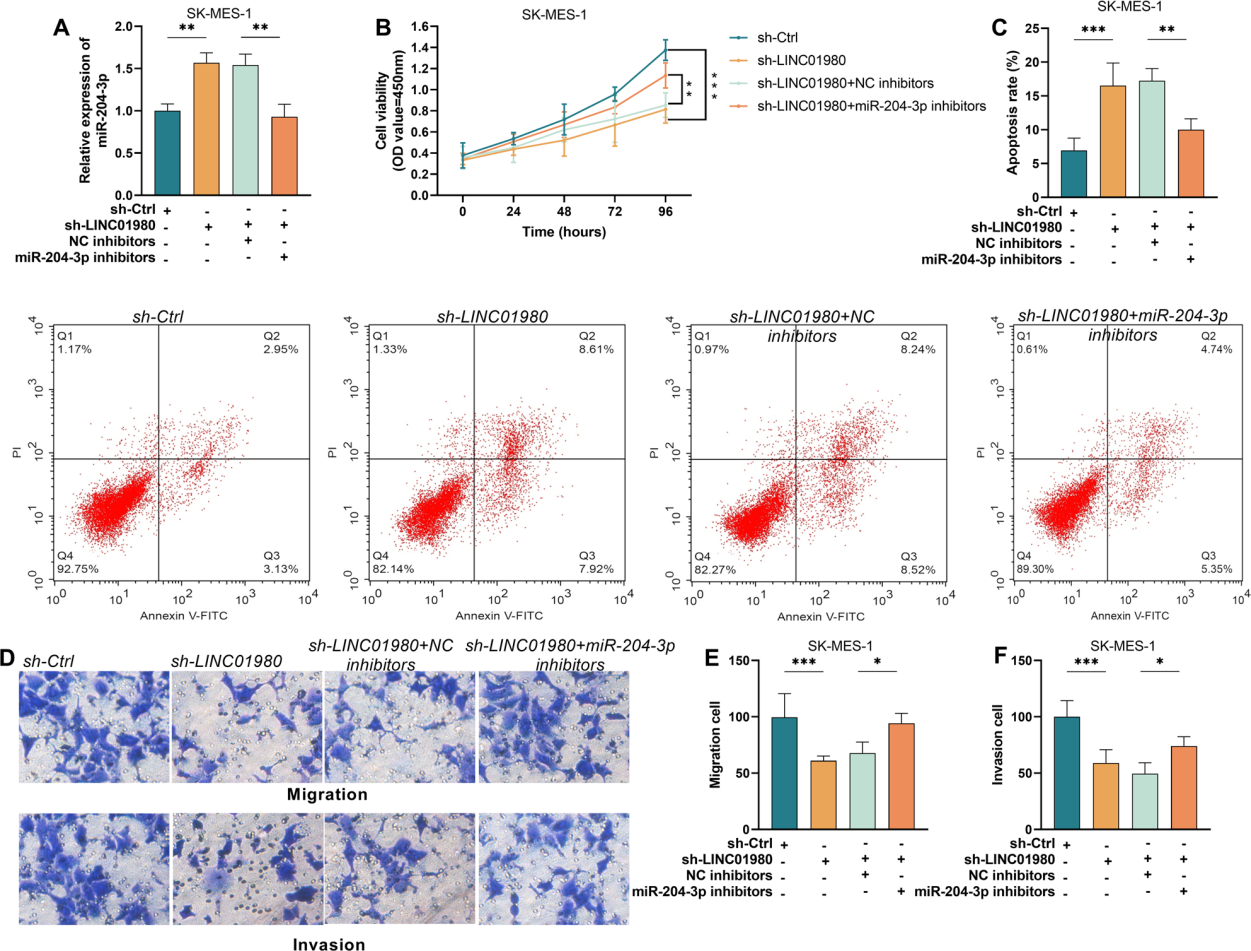
LINC01980 modulates the malignant biological behaviors of lung squamous carcinoma cells via targeting miR-204-3p. **A**. Following the downregulation of LINC01980 to inhibit miR-204-3p, the expression level of miR-204-3p was assessed using RT-qPCR. **B**. The viability of SK-MES-1 cells was evaluated through a CCK-8 assay. **C**. Apoptosis and apoptosis rates in SK-MES-1 cells under different transfection conditions were analyzed by flow cytometry. **D**. The migration and invasion capacities of LUSC cells under different transfection conditions were evaluated using transwell assays. Statistical analysis of SK-MES-1 cell migration (**E**) and invasion (**F**) rates. * P < 0.05, ** P < 0.01, *** P < 0.001

To clarify the functional mechanism of miR-204-3p in the regulation of LUSC progression by LINC01980, we performed a rescue assay. Experimental data showed that miR-204-3p levels were significantly up-regulated when the expression of LINC01980 was inhibited in SK-MES-1 cells, but this effect was significantly suppressed by simultaneous transfection with miR-204-3p inhibitor (*P* < 0.01, Fig. 5A). It was further found that the impaired cell proliferation ability and increased apoptosis caused by LINC01980 knockdown were largely eliminated after the miR-204-3p expression was inhibited (*P* < 0.01, Fig. 5B-C). Inhibition of miR-204-3p was shown to significantly reverse the impaired cell migration and invasion ability due to LINC01980 downregulation by Transwell analysis (*P* < 0.05, Fig. 5D-F). Overall, these outcomes solidify the role of LINC01980 in altering the malignant biological traits of LUSC cells through the regulation of miR-204-3p expression.

## Discussion

Lung cancer accounts for a large proportion of cancer-related deaths worldwide [16, 17], and among lung cancers, LUSC has a particularly high risk of metastasis and recurrence [18]. Several studies have shown that this type of tumor is characterized by great heterogeneity [19, 20] and is likely to develop resistance to existing therapeutic options [21]. Despite recent significant advances in targeted therapies and immunotherapy [22], there are still no effective targeted therapies for LUSC, preventing improved patient survival. Therefore, the systematic elucidation of the pathogenesis of LUSC and the advancement in new biomarkers and therapeutic endpoints will be very useful for improving clinical diagnosis and treatment.

A substantial and expanding body of literature demonstrates the indispensable functions of LncRNAs in some malignancies, including LUSCs. For example, the up-regulated expression of BBOX1-AS1 in LUSC tissues suggests an oncogenic role [23]. LncRNA RP11-116G8. 5 increases PHF12/FOXP4 expression to promote the malignant behavior of LUSCs [24]. LINC01980, as a lncRNA newly discovered in recent years, is aberrantly expressed in multiple neoplastic conditions, such as esophageal [25], gastrointestinal [26], and colorectal cancers [27], and is involved in tumor proliferation and metastasis. Recent studies have revealed its aberrant expression in squamous lung cancer [11, 12], but its role has not been fully elucidated. After analyzing clinical samples and LUSC cell lines, we found that LINC01980 expression levels were significantly elevated, which could increase the proliferative capacity and migratory invasiveness of tumor cells and inhibit the apoptotic process. The biological functions of lncRNAs may depend on differences in localization. We found that LINC01980 is primarily distributed in the cytoplasm. Cytoplasmically located lncRNAs can interact with miRNAs in the form of competitive endogenous RNA (ceRNA) to regulate the expression of downstream target genes of these miRNAs. To determine whether LINC01980 functions as a ceRNA in LUSCs via a miRNA-binding mechanism, we used bioinformatic predictions combining a reporter system utilizing dual luciferases and RNA extraction assays, and our results indicate that miR-204-3p may be the target of action of LINC01980. As important post-transcriptional regulators, miRNAs play an important role in the development of LUSC. miR-204-3p is encoded by the miR204 gene, located on human chromosome 9, region 9q34.13. Previous studies have shown that this molecule can suppress the development of NSCLC [15, 28] and LUAD [29]. Nevertheless, the specific biological function of miR-204-3p in LUSCs remains unclear.

The experimental data from this study showed significant downregulation of miR-204-3p expression in LUSC tumor tissues and cellular models, and it targets and binds to LINC01980. A body of evidence indicates that miR-204-3p functions as a tumor suppressor, suggesting that LINC01980 is likely to influence LUSC development by regulating miR-204-3p expression. However, this study still has shortcomings: firstly, the results obtained in a larger clinical cohort need to be validated, and secondly, the experiments were primarily conducted using cell lines and clinical tissue samples, without in vivo animal validation. Future studies will employ nude mouse tumor xenograft models to further validate the biological functions and therapeutic potential of the LINC01980/miR-204-3p axis. Additionally, this study has not fully elucidated the downstream target genes of miR-204-3p or the specific signaling pathways it regulates. In further studies, we will identify direct effector molecules of miR-204-3p and employ bioinformatics approaches, including the GO-based functional annotation and KEGG-based pathway analysis, to thoroughly map the molecular regulation of LINC01980/miR-204-3p in LUSC. The predicted results will be validated through experimental methods.

In conclusion, this study reveals the critical role of LINC01980 in LUSC. Based on its function as a micro-sponge for miR-204-3p, future research may explore multiple intervention strategies for LUSC patients. For instance, antisense oligonucleotides (ASOs) could be designed to specifically degrade LINC01980 transcripts, or CRISPRi technology could target its promoter region to suppress expression. Future research will focus on validating the efficacy and safety of these intervention strategies in preclinical models, aiming to develop novel therapeutics for LUSC.

## Conclusion

In this study, we have shown that LINC01980 regulates and participates in the malignant transformation of LUSC by targeting miR-204-3p, and that high LINC01980 expression is significantly associated with accelerated disease progression. These results suggest that LINC01980 could be a novel molecular marker and therapeutic target for the treatment of LUSC.

## Data Availability

All data generated or analyzed during this study are included in this article. Further enquiries can be directed to the corresponding author.

## Abbreviations

NSCLC: Non-small cell lung cancer
LncRNAs: Long noncoding RNAs
CeRNA: Competitive endogenous RNA
FBS: Fetal bovine serum
PBS: Phosphate buffer solution
MUT: Mutant
NC: Negative control
